# Leiomyoma of the Breast: A Report of a Rare Case of a Benign Breast Tumor From the Caribbean

**DOI:** 10.7759/cureus.80678

**Published:** 2025-03-16

**Authors:** Parvati Raghunath, Ravi Maharaj, Dave Harnanan, Shaheeba Barrow, Vijay Naraynsingh

**Affiliations:** 1 Surgery, Medical Associates Hospital, St. Joseph, TTO; 2 Surgical Sciences, The University of the West Indies, St. Augustine, TTO; 3 Clinical Surgical Sciences, The University of the West Indies, St. Augustine, TTO; 4 Pathology, Port of Spain General Hospital, Port of Spain, TTO

**Keywords:** breast fibroid, breast leiomyoma, breast neoplasm, rare benign breast tumour, surgical management of breast leiomyoma, tumour resection with free margins

## Abstract

Leiomyomas are benign mesenchymal stromal tumors of smooth muscle; while these are more traditionally found in the uterus, in rare cases, they can also be found in the breast, particularly in the subareolar region. Clinically and radiographically, these tumors are very similar to fibroadenomas but can be definitively distinguished based on distinctive histopathological features. Surgical management for breast leiomyomas has previously included procedures ranging from lumpectomy to mastectomy; however, resection of the tumor with free margins is the most well-recognized treatment for breast leiomyomas.

## Introduction

Leiomyomas, more colloquially known as “fibroids”, are usually well-known to be found in the uterus and can sometimes be seen in the gastrointestinal tract, skin, and lower extremities; however, these tumors can occur in the breast in rare cases, with fewer than 30 cases reported so far, and accounts for less than 1% of all breast neoplasms [1]. Breast leiomyomas usually involve the right breast and are most commonly seen in women aged 30-60 years, with an average age of onset of 47.5 years [1]. We report a case of this rare entity in a 74-year-old patient.

## Case presentation

**Figure 1:**
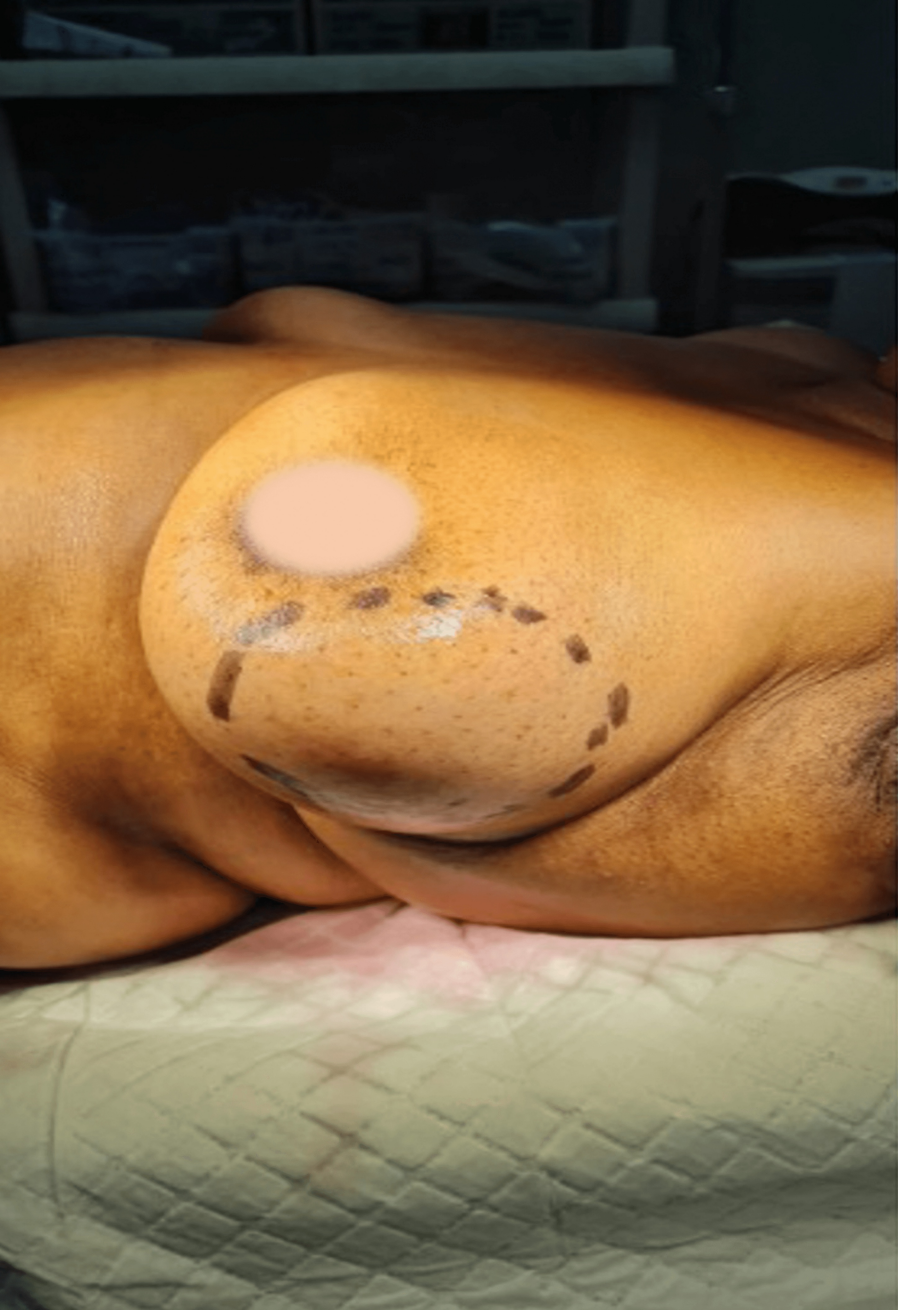
Demarcated area showing mass with well-defined edges (areolar blurred)

A 74-year-old female presented to our care with a painless 12 cm lump in the left breast that had enlarged slowly over the preceding three years. On examination, we observed a freely mobile mass with well-defined edges, a very smooth surface, and soft to firm in consistency (Figure 1). Clinically, it was benign, and the margins were clearly defined on ultrasound. Fine needle aspiration cytology (FNAC) showed no evidence of malignancy.

**Figure 2:**
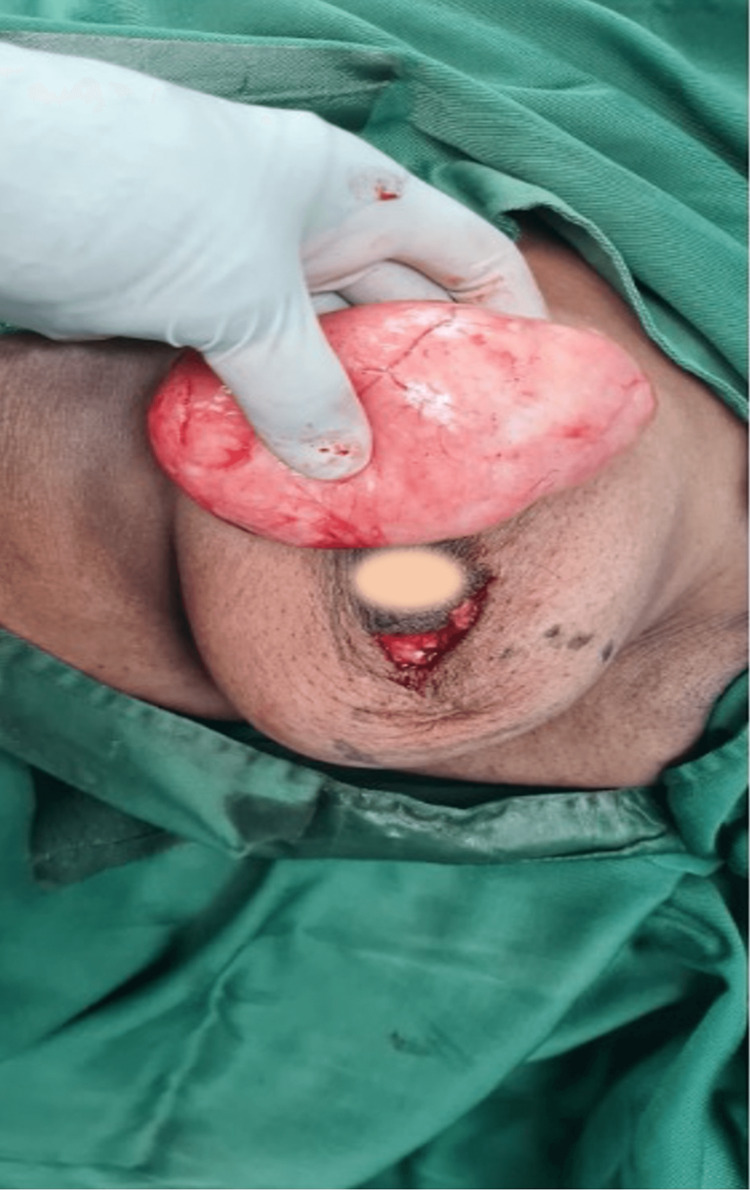
Circumareolar incision made during surgery The mass is mobilized by blunt resection with the finger and delivered through the incision. The mass had a smooth surface and was soft to firm in consistency (areolar blurred)

During surgery, a circumareolar incision was made (Figure 2) and the mass was mobilized by blunt resection with the finger. It was very smooth, and we found it easy to peel off the surrounding tissue. Because it was soft and deformable, it was possible to deliver it through an incision that was a bit smaller than the mass itself.

**Figure 3:**
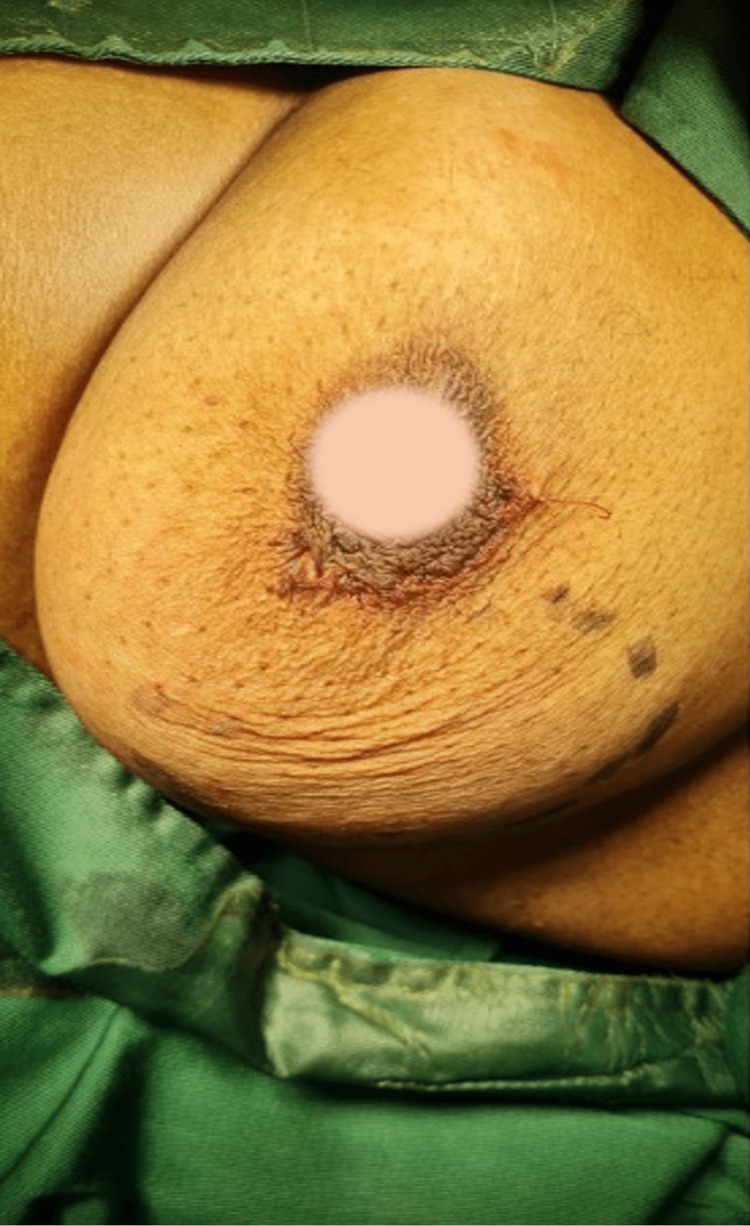
Closure done with subcuticular vicryl suture (areolar blurred)

A subcuticular vicryl suture was used for closure (Figure 3), and the patient recovered uneventfully. The histology later showed a leiomyoma.

## Discussion

Leiomyomas are benign mesenchymal stromal tumors of smooth muscle that are very commonly seen in the uterus, gastrointestinal tract, skin, and lower extremities; however, they can present in the breast in rare cases [1]. When they occur in the breast, leiomyomas are typically found superficially, in the subareolar region, where there is more smooth muscle, or, less commonly, deep in the breast parenchyma [2]. With only a few cases of intraparenchymal leiomyoma of the breast reported to date, this condition is exceedingly rare, accounting for less than 1% of all breast neoplasms [1]. There have been no prior reports of leiomyomas of the breast from the Caribbean, making this case, to our knowledge, the first of its kind to be reported in this region.

Breast Leiomyomas usually involve the right breast and are ordinarily seen in women aged 30-60 years, with an average age of onset of 47.5 years [1]. Only one case has been recorded in a male so far [3]. Our patient was 74 years old. There has been no definitive explanation for the origin of this tumor, although many theories exist. Kauffman et al. have proposed that this tumor stems from the myocytes that environ the capillaries in the subcutaneous tissues in the mammary glands [4], while Diaz-Arias et al. [5] suggest five sources of origin: teratoid origin joint with overgrowth of myomatous elements; smooth muscle cells that were displaced from the nipple during embryological development; a multipotent mesenchymal cell; angiomatous smooth muscle; and myoepithelial cells.

Clinically, breast leiomyomas are typically firm, have a fibroelastic consistency, or are sometimes hard, with well-defined limits. They may present as mobile or non-mobile lumps and patients can experience intermittent pain or no pain, without any nipple discharge [6,7,8]. These breast leiomyomas come with prior mammographic evidence of being well-circumscribed, isodense or hypodense masses, while on sonography, the tumor would appear to be well-circumscribed, ellipsoid shaped, and hypoechoic [9]. Since these findings are rather similar to those of a fibroadenoma, with the only difference being the absence of distal attenuation [5,10], the distinction is primarily made by histology. These tumors have histopathological features that are identical to leiomyomas of other sites. These include eosinophilic cytoplasm and groups of intertwining bundles of spindle-shaped cells with blunt end nuclei [4]. They are also positive for desmin, vimentin, and muscle-specific actin on immunoperoxidase staining [5].

Surgical management for breast leiomyomas has previously included procedures ranging from lumpectomy to mastectomy; however, the most well-recognized treatment for breast leiomyomas involves THE resection of the tumor with free margins [11]. This contrasts with the surgical management of a main malignant differential for breast leiomyomas, and leiomyosarcomas, which require surgical resection with negative margins [12].

## Conclusions

We reported a case of leiomyoma of the breast in an elderly patient who was surgically managed. Our findings suggest that the preoperative confirmation of the benign nature of the disease histologically makes it safe to perform the excision through a very limited circumareolar incision and deliver the lump from wherever in the breast it is found since it can be mobilized and delivered through this incision.
